# Localization of peroxisome proliferator-activated receptor alpha (PPARα) and *N*-acyl phosphatidylethanolamine phospholipase D (NAPE-PLD) in cells expressing the Ca^2+^-binding proteins calbindin, calretinin, and parvalbumin in the adult rat hippocampus

**DOI:** 10.3389/fnana.2014.00012

**Published:** 2014-03-17

**Authors:** Patricia Rivera, Sergio Arrabal, Antonio Vargas, Eduardo Blanco, Antonia Serrano, Francisco J. Pavón, Fernando Rodríguez de Fonseca, Juan Suárez

**Affiliations:** ^1^Laboratorio de Investigación (UGC Salud Mental), Instituto de Investigación Biomédica (IBIMA), Universidad de Málaga-Hospital Regional Universitario de MálagaMálaga, Spain; ^2^CIBER OBN, Instituto de Salud Carlos III, Ministerio de Ciencia e InnovaciónMadrid, Spain; ^3^Departament de Pedagogia i Psicologia, Facultat de Ciències de l'Educació, Universitat de LleidaLleida, Spain

**Keywords:** PPAR alpha, NAPE-PLD, calcium-binding protein, hippocampus, rat, immunohistochemistry, confocal microscopy

## Abstract

The *N*-acylethanolamines (NAEs), oleoylethanolamide (OEA) and palmithylethanolamide (PEA) are known to be endogenous ligands of PPARα receptors, and their presence requires the activation of a specific phospholipase D (NAPE-PLD) associated with intracellular Ca^2+^ fluxes. Thus, the identification of a specific population of NAPE-PLD/PPARα-containing neurons that express selective Ca^2+^-binding proteins (CaBPs) may provide a neuroanatomical basis to better understand the PPARα system in the brain. For this purpose, we used double-label immunofluorescence and confocal laser scanning microscopy for the characterization of the co-existence of NAPE-PLD/PPARα and the CaBPs calbindin D28k, calretinin and parvalbumin in the rat hippocampus. PPARα expression was specifically localized in the cell nucleus and, occasionally, in the cytoplasm of the principal cells (dentate granular and CA pyramidal cells) and some non-principal cells of the hippocampus. PPARα was expressed in the calbindin-containing cells of the granular cell layer of the dentate gyrus (DG) and the SP of CA1. These principal PPARα^+^/calbindin^+^ cells were closely surrounded by NAPE-PLD^+^ fiber varicosities. No pyramidal PPARα^+^/calbindin^+^ cells were detected in CA3. Most cells containing parvalbumin expressed both NAPE-PLD and PPARα in the principal layers of the DG and CA1/3. A small number of cells containing PPARα and calretinin was found along the hippocampus. Scattered NAPE-PLD^+^/calretinin^+^ cells were specifically detected in CA3. NAPE-PLD^+^ puncta surrounded the calretinin^+^ cells localized in the principal cells of the DG and CA1. The identification of the hippocampal subpopulations of NAPE-PLD/PPARα-containing neurons that express selective CaBPs should be considered when analyzing the role of NAEs/PPARα-signaling system in the regulation of hippocampal functions.

## Introduction

*N*-acylethanolamines (NAEs), including oleoylethanolamide (OEA) and palmithylethanolamide (PEA), exert a variety of biological activities in the central nervous system (Calignano et al., 1998; Combs et al., 2001; Inoue et al., 2001; Rodríguez de Fonseca et al., 2001; Okamoto et al., 2007). NAE biosynthesis has been proposed to occur on demand via a two-step enzymatic process. First, a Ca^2+^-activated *N*-acyltransferase transfers the *ns*-1 acyl chain of a phospholipid to the amine of phosphatidylethanolamine (PE) to generate an *N*-acyl PE (NAPE) (Di Marzo et al., 1994). Then, NAPE is converted into an NAE and phosphatidic acid by phospholipase D (PLD) (Schmid, 2000). The molecular identity of the Ca^2+^-activated *N*-acyltransferase is currently unknown, while the *N*-acyl phosphatidylethanolamine phospholipase D (NAPE-PLD) that is expressed in specific regions of the brain has been identified (Di Marzo et al., 1996; Okamoto et al., 2004). The brain tissue of NAPE-PLD knockout mice showed a five-fold reduction in the Ca^2+^-dependent conversion of NAPEs to NAEs, which affected both endocannabinoids, such as anandamide, and the PPARα receptor ligands PEA and OEA (Leung et al., 2006). Accordingly, NAPE-PLD activity is highly associated with intracellular Ca^2+^ stores in several types of hippocampal excitatory axon terminals (Nyilas et al., 2008).

The PPARα receptor, which shows specific patterns of localization in the brain (Braissant et al., 1996; Cullingford et al., 1998; Moreno et al., 2004), appears to mediate the signaling effects of monounsaturated NAEs. Its activation results in the up-regulation of target genes involved in learning, memory, aging, neurodegeneration and inflammation (Combs et al., 2001; Inoue et al., 2001). Several reports showed that PPARα activation resulted in prevention of cognitive impairment (Greene-Schloesser et al., 2014) and neuroprotection after neuronal damage in animal models of Parkinson's diseases or after excitotoxic lesions and ischemia (Lombardi et al., 2007; Sun et al., 2007; Bisogno and Di Marzo, 2008; Galán-Rodríguez et al., 2009; Garg et al., 2011; Koch et al., 2011; Esposito et al., 2012; Zhou et al., 2012). Moreover, PPARα activation induces biological mechanisms that require the participation of intracellular Ca^2+^ (Di Marzo et al., 1994; Khasabova et al., 2012), such as the induction of peroxisomal proliferation, attenuation of neurotoxicity, decreases in intraneuronal ROS production and prevention of the calcium influx induced by H_2_O_2_ (Santos et al., 2005; Galán-Rodríguez et al., 2009; Esposito et al., 2012; Scuderi et al., 2012).

Calcium-binding proteins (CaBPs) belonging to the calmodulin superfamily play important roles as intracellular Ca^2+^ buffers and sensors in mediating Ca^2+^-dependent events, such as synaptic transmission and axonal transport (Nakamura et al., 1980). Several CaBPs, including calbindin D28k, calretinin and parvalbumin, have been found in high concentrations in the brain (Baimbridge et al., 1982; García-Segura et al., 1984). CaBPs deficits have been related to relevant neurodegenerative processes, such as Alzheimer's disease, Parkinson's disease, age-related cognitive defects, schizophrenia, epilepsy, and some forms of tumors (Maglóczky et al., 1997; Cates et al., 2006; Nakazawa et al., 2012; Verret et al., 2012). These CaBPs usually correlate with neurotransmitter content, cell morphology, distribution, and function (Baimbridge et al., 1982; Celio, 1990; Gulyás et al., 1991) and are used to classify neurons into specific subpopulations. For instance, hippocampal non-pyramidal cells containing calretinin and parvalbumin are usually GABAergic neurons (Kosaka et al., 1987; Miettinen et al., 1992; Wouterlood et al., 2001). Moreover, it was demonstrated that parvalbumin-positive CA1 interneurons are required for spatial working but not for reference memory (Murray et al., 2011), whereas calbindin-positive granule cells of the dentate gyrus (DG) contribute to verbal memory impairments in temporal lobe epilepsy (Karádi et al., 2012).

It has been hypothesized that CaBPs buffer the intracellular Ca^2+^ levels, resulting in the regulation of NAE production through the Ca^2+^-sensitive enzyme NAPE-PLD, and as a consequence, CaBPs can influence PPARα activity. This hypothesis supports the need for the identification of the cells that co-express or do not co-express the CaBPs and the NAPE-PLD/PPARα signaling system. In the present study, we described and systematically characterized specific hippocampal PPARα and NAPE-PLD-containing cells that express the three CaBPs, calbindin D28k, calretinin, and parvalbumin. For this purpose, we used double-label immunofluorescence and confocal laser scanning microscopy.

## Materials and methods

### Animals

Adult male Wistar rats (*n* = 5), weighing approximately 250 g and 10–12 weeks old (Charles River Laboratories, Barcelona, Spain), were used in this study. Male total-NAPE-PLD-KO mice and male total-PPARα-KO mice (*n* = 2), weighing approximately 25 g and 8 weeks old, were used in this study. The animals were kept in standard conditions (Servicio de Estabulario, Facultad de Medicina, Universidad de Málaga) at 20 ± 2°C room temperature, 40 ± 5% relative humidity and a photoperiod of 12L:12D; the rats were given free access to food and water. All experimental animal procedures were performed in compliance with the European Communities directive 86/609/ECC and Spanish legislation (BOE 252/34367-91, 2005) regulating animal research.

### Tissue processing

The animals were anesthetized with sodium pentobarbital (50 mg/kg, i.p.) and transcardially perfused with 0.1 M phosphate-buffered saline (PBS; pH 7.3), followed by 4% formaldehyde in PBS. The brains were dissected and incubated in the same fixative solution overnight at 4°C, then cryoprotected in 0.1 M phosphate-buffered saline pH 7.3 (PBS) containing 30% sucrose and 0.01% sodium azide (NaN_3_) for 48 h. Then, the brains were cut into 30-μ m thick transverse sections using a sliding microtome. The sections were stored at 4°C in PBS containing 0.002% (w/v) NaN_3_ until immunohistochemistry analysis.

### Immunohistochemistry

For the analysis of the immunohistochemical expression of PPARα, NAPE-PLD and the Ca^2+^-binding proteins (calbindin, calretinin, and parvalbumin) in the hippocampus, free-floating, 30-μ m thick coronal sections from the −3.00 to −4.80 mm Bregma levels were used (Paxinos and Watson, 2007). The sections were first washed several times with 0.1 M PBS (pH 7.3) to remove the NaN_3_ and were incubated in H_2_O containing 50 mM sodium citrate (pH 6) for 30 min at 80°C, followed by several washes in 0.1 M PBS (pH 7.3). Then, the sections were incubated in a solution of 3% hydrogen peroxide and 10% methanol in 0.1 M PBS for 20 min at room temperature in the dark to inactivate the endogenous peroxidase, followed by washes in PBS. The sections were then blocked with 10% donkey or goat serum in PBS containing 0.1% NaN_3_ and 0.2% Triton X-100 and incubated with a primary antibody overnight at room temperature (for details regarding the antibodies used, see Tables 1, 2).

**Table 1:** **Primary antibodies used**.

**Antigen**	**Immunogen**	**Manufacturing details**	**Dilution**	**References**
PPARα	Synthetic Peptide: M(1)VDTESPICPLSPLEADD (18)C	Fitzgerald	1:100	Suardíaz et al., 2007
		Affinity purified polyclonal IgG antibody		
		Developed in rabbit		
		Code No.: 20R-PR021		
		Lot. No.: P11120812		
NAPE-PLD	Mouse N-terminal 1-41aa polypeptide (AB112350): MDEYEDSQSPAPSYQYPKETLRKR QNSVQNSGGSVSSRFSR	Frontier Institute	1:500	Leung et al., 2006 Nyilas et al., 2008
		Affinity purified polyclonal IgG antibody		
		Developed in guinea pig		
		Code No. GP-Af720		
		Lot. No.: Not provided		
Calbindin	Calbindin D28k purified from chicken gut: MTAETHLQGVEISAAQFFEIWHHYDSDG NGYMDGKELQNFIQELQQARKKAGLDL TPEMKAFVDQYGKATDGKIGIVELAQVL PTEENFLLFFRCQQLKSSEDFMQTWRKY DSDHSGFIDSEELKSFLKDLLQKANKQIE DSKLTEYTEIMLRMFDANNDGKLELTEL ARLLPVQENFLIKFQGVKMCAKEFNKAF EMYDQDGNGYIDENELDALLKDLCEKN KKELDINNLATYKKSIMALSDGGKLYRA ELALILCAEEN	Swant	1:500	Celio, 1990 Rüttimann et al., 2004 Suárez et al., 2005
		Monoclonal IgG antibody		
		Produced in mouse myeloma cells		
		Code No.: 300		
		Lot. No.: 07 (F)		
Calretinin	Recombinant human calretinin 22k (epitope within the first 4 EF-hands domains): MAGPQQQPPYLHLAELTASQFLEIWKHF DADGNGYIEGKELENFFQELEKARKGSG MMSKSDNFGEKMKEFMQKYDKNSDGK IEMAELAQILPTEENFLLCFRQHVGSSAE FMEAWRKYDTDRSGYIEANELKGFLSDL LKKANRPYDEPKLQEYTQTILRMFDLNG DGKLGLSEMSRLLPVQENFLLKFQGMKL TSEEFNAIFTFYDKDRSGYIDEHELDALL KDLYEKNKKEINIQQLTNYRKSVMSLAE AGKLYRKDLEIVLCSEPPM	Swant	1:500	Zimmermann and Schwaller, 2002 Rüttimann et al., 2004 Suárez et al., 2006
		Monoclonal antibody		
		Developed in mouse		
		Code No.: 6B3		
		Lot. No.: 010399		
Parvalbumin	Parvalbumin purified from carp muscles: MAFAGILNDADITAALQGCQAADSFDY KSFFAKVGLSAKTPDDIKKAFAVIDQDK SGFIEEDELKLFLQNFSAGARALTDAETK AFLKAGDSDGDGKIGVDEFAALVKA	Swant	1:500	Celio, 1986 Bouilleret et al., 2000
		Monoclonal IgG antibody		
		Produced in mouse myeloma cells		
		Code No. 235		
		Lot. No.: 10–11 (F)		

**Table 2:** **Secondary antibodies used**.

**Antigen**	**Produced in**	**Conjugate to**	**Manufacturing details**	**Dilution**
Anti-rabbit IgG	Donkey	Biotin	GE Healthcare	1:500
			Code No.: RPN1004	
			Lot. No.: 5356499	
Anti-mouse IgG	Goat	Biotin	SIGMA	1:500
			Code No.: B 7264	
			Lot. No.: 125K6063	
Anti-guinea pig IgG	Goat	Biotin	Vector Laboratories	1:500
			Code No.: BA-7000	
			Lot. No.: W0726	
Anti-rabbit IgG	Donkey	Cy3 bis-NHS ester	Jackson ImmunoResearch	1:300
			Code No.: 711-166-152	
			Lot. No.: 101675	
Anti-mouse IgG	Goat	Fluorescein Isothiocyanate (FITC)	SIGMA	1:300
			Code No.: F2012	
			Lot. No.: 107K6058	
Anti-guinea pig IgG	Goat	Cy3 bis-NHS ester	Jackson	1:300
			ImmunoResearch	
			Code No.: 106-165-003	
			Lot. No.: 106592	

The following day, the sections were washed in PBS and incubated with a biotinylated secondary antibody diluted 1:500 for 1 h (Table 2). The sections were washed again in PBS and incubated with a 1:2000 dilution of ExtrAvidin peroxidase (Sigma, St. Louis, MO) for 1 h. After several washes, immunolabeling was revealed by exposure to 0.05% diaminobenzidine (DAB; Sigma), 0.05% nickel ammonium sulfate and 0.03% H_2_O_2_ in PBS. After several washes in PBS, the sections were mounted on slides treated with poly-l-lysine solution (Sigma), air-dried, dehydrated in ethanol, cleared with xylene and coverslipped with Eukitt mounting medium (Kindler GmBH & Co, Freiburg, Germany). Digital high-resolution photomicrographs of the rodent brains were taken under the same conditions of light and brightness/contrast using an Olympus BX41 microscope equipped with an Olympus DP70 digital camera (Olympus Europa GmbH, Hamburg, Germany).

### Double immunofluorescence

Hippocampal sections were pretreated as described above and incubated overnight at room temperature with a cocktail of primary antibodies (Table 1). After washing in 0.1 M PBS (pH 7.3), the sections were incubated for 2 h at room temperature with a cocktail of fluorescent secondary antibodies (Table 2) for 2 h. In some cases, we used the nuclei marker 4',6-diamine-2-phenylindole dihydrochloride (DAPI, ref. no. D9542, SIGMA) to identify the cell nuclei of specific hippocampal cell populations. For epifluorescence analysis, digital high-resolution microphotographs were taken using an Olympus BX41 fluorescence microscope equipped with an Olympus DP70 digital camera (Olympus). For a more detailed analysis, the sections that were doubly labeled were visualized using a confocal laser (spectral) scanning microscope (Leica TCS NT; Leica Microsystems) equipped with a 561 nm DPM laser (argon 30%) and a 63 × objective (HCX PL APO CS 63.0×1.40 OIL UV). The numerical aperture was 1.40. The emission filter settings were 430–483 nm for PMT1 (blue), 504–545 nm for PMT2 (green), and 570–630 nm for PMT3 (red). The channels of the images were taken sequentially with a frame average of 3. Depending on the level of zoom used in each image, the XY voxel size ranged from 240.5 nm (zoom = 1) to 29.4 nm. The pinhole (airy) was 1. The section thickness (*Z*) was 772 nm. Thus, we could discriminate the labeling of those structures whose size was larger than the image resolution. Settings of light and brightness/contrast were adjusted by using the Leica LAS AF Lite imaging software.

### Antibody specific and controls

We performed Western blot analyses to demonstrate that the PPARα, NAPE-PLD, calbindin, calretinin, and parvalbumin antibodies recognized the corresponding antigen in the rat hippocampus. To perform Western blot analysis, we used fresh tissue from Wistar male rats. The animals were sacrificed using 2,2,2-tribromoethanol (Fluka, Steinheim, Germany), and the hippocampi were immediately isolated, snap frozen in liquid nitrogen and stored at −80°C until use. Protein extracts of the rat hippocampi were prepared in RIPA buffer (50 mM Tris-HCl pH 7.4, 150 mM NaCl, 0.25% NaDOC, 1% Triton-X100, 1 mM EDTA, 10% aprotinin) using a homogenizer. After 2 h of incubation with agitation at 4°C, the homogenate was centrifuged at 20,800 g for 20 min at 4°C, and the supernatant was collected.

**Figure 1:**
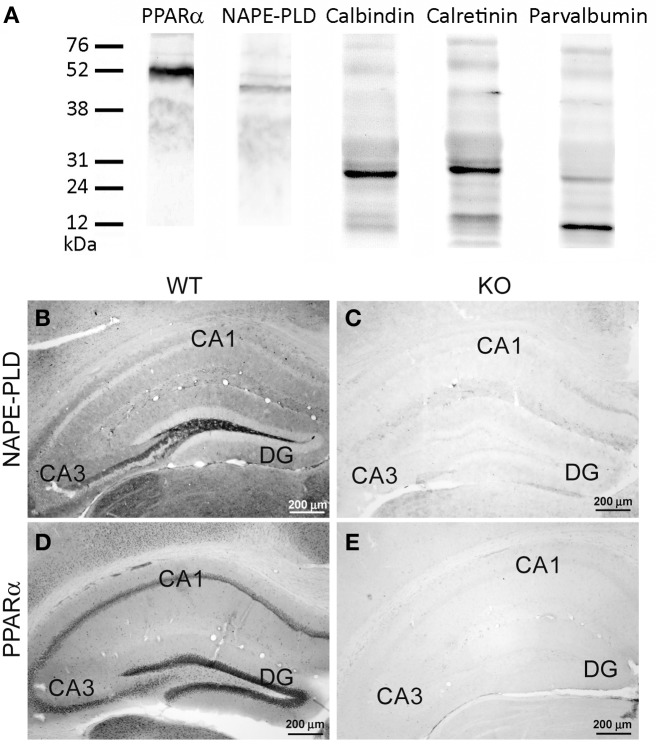
**(A)** Western blot analysis of protein extracts from the rat hippocampus showing the prominent immunoreactive bands of the expected molecular masses of 52 kDa for PPARα, 46 kDa for NAPE-PLD, 28 kDa for calbindin, 29 kDa for calretinin, and 10 kDa for parvalbumin. The positions of the molecular markers are indicated on the left. **(B,C)** Comparative immunohistochemical analysis of NAPE-PLD expression in the hippocampus (DG, CA1, CA3) of the NAPE-PLD wild-type mouse **(B)** and the NAPE-PLD knockout male mouse **(C)**. **(D,E)** Comparative immuno- histochemical analysis of PPARα expression in the hippocampus (DG, CA1, CA3) of the PPARα wild-type mouse **(D)** and the PPARα knockout male mouse **(E)**. For abbreviations, see Table 3 legend. Scale bars are indicated in each image.

For immunoblot analysis, equivalent amounts of protein extract (75 μg) were separated on 4–20% precast polyacrylamide gels (Criterion™ TGX™ Precast Gel, Bio-Rad, cat. no. 567-1093), electroblotted onto nitrocellulose membranes and stained with Ponceau red to ensure equal loading. The blots were first incubated with a blocking buffer containing 2% bovine serum albumin (Merck) in PBS and 0.1% Tween 20 at room temperature for 1 h. Then, each blotted membrane lane was incubated separately with the specific PPARα (1:500), NAPE-PLD (1:200), calbindin D28k (1:500), calretinin (1:1000), or parvalbumin (1:1000) antibodies. Peroxidase-conjugated goat anti-rabbit, goat anti-mouse, and goat anti-guinea pig antibodies (dilution 1:2000; Promega, Madison, WI, USA) were added for 1 h at room temperature. The specific protein bands were visualized using the enhanced chemiluminescence technique (ECL, Amersham) and the Auto-Biochemi Imaging System (LTF Labortechnik GmbH, Wasserburg/Bodensee, Germany). Western blot analysis showed that each primary antibody detected a protein of the expected molecular size (Figure 1A).

Because of the described variability of NAPE-PLD distribution with respect to the different antibodies used (Egertová et al., 2008; supplementary figures in Suárez et al., 2008; present study), we carried out additional control experiments to ensure the specificity of the antibodies. To this end, the hippocampus of a wild-type mouse was compared with those of NAPE-PLD knock-out mice (Leung et al., 2006; Nyilas et al., 2008). The specificity of the PPARα antibody in the brains of the PPARα knock-out mice was also tested (Suardíaz et al., 2007). The immunohistochemical protocol for NAPE-PLD and PPARα was carried out as described above (Table 1). We observed that immunostaining was completely absent in the hippocampus from the NAPE-PLD knock-out mouse (Figures 1B,C) and PPARα knock-out mouse (Figures 1D,E) when compared with those of the respective wild-type mice (see references in Table 1 for further information).

Calbindin D28k, calretinin, and parvalbumin antibodies were evaluated for specificity and potency (see references in Table 1) using several methods: (a) by indirect immunofluorescent or immunoperoxidase labeling, as well as biotin-avidin labeling, of 4% formaldehyde fixed brains; (b) by immunoenzymatic labeling of immunoblots; (c) by radioimmunoassay; or (d) by immunohistochemistry of the brain tissue of calbindin knock-out mice, calretinin knock-out mice or parvalbumin knock-out mice, respectively.

## Results

In the present study, we first analyzed the distribution and the co-expression of PPARα and NAPE-PLD with the CaBPs calbindin, calretinin, and parvalbumin in the rat hippocampus. The intensity of the immunoreactivity for each antibody was similar in all brains analyzed in the present study. The results for this study are described in the text and are summarized using a rating scale (Table 3). The gray-scale values measured in the hippocampus are represented using an arbitrary scale of three labeling intensities, from “+” meaning “low” (above the background density) to “+++” meaning “high” (according to the highest signal density in the specimen). Previously, we performed Western blot analysis to ensure that the PPARα, NAPE-PLD, calbindin, calretinin, and parvalbumin antibodies recognized the corresponding antigens in the rat hippocampus (Figure 1A). Thus, Western blot analyses of the protein extracts from the rat hippocampus revealed PPARα immunostaining as a prominent band at approximately 52 kDa. Immunoblots for NAPE-PLD also revealed a single band with a molecular mass of 46 kDa. Analysis of calbindin D28k, calretinin and parvalbumin confirmed the expected bands of 28, 29, and 10 kDa, respectively (Figure 1A).

**Table 3:** **Immunoreactivity in the rat hippocampus^a^**.

		**PPARα**	**NAPE-PLD**		**Calbindin**		**Calretinin**		**Parvalbumin**	
		**Nuclei**	**Somata**	**Fibers**	**Somata**	**Fibers**	**Somata**	**Fibers**	**Somata**	**Fibers**
DG	ml	+	−	+	+	+++	−	+	−	−
	gcl	+++	+	−	+++	+++	−	−	+	+++
	pcl	+	++	−	+	+	+	+	−	−
CA3	SO	+	+	+	−	−	−	+	−	+
	SP	+++	++	+	−	−	+	+	++	+++
	SR	+	−	−/+ (SL)	+	−/+++ (SL)	+	+	−	+
CA1	SO	+	−	+	+	−	−	−	+	−
	SP	+++	+	+	+	−	+	−	+	++
	SR	+	+	−	+	−	+	−	−	−
	SL-M	+	+	+	+	+	+	−	−	−

a Rating scale of the immunoreactivity in nuclei, somata and fibers of each layer and stratum of the hippocampus. Symbols are as follows: high (+++), medium (++), low (++), and without immunoreactivity (−). Abbreviations: DG, dentate gyrus; gcl, granular cell layer; ml, molecular layer; pcl, polymorphic cell layer (hilus); SL, stratum lucidum; SL-M, stratum lacunosum-moleculare; SO, stratum oriens; SP, stratum pyramidale; SR, stratum radiatum.

### Distribution of PPARα, NAPE-PLD, and CaBPs in the adult rat hippocampus

**Figure 2:**
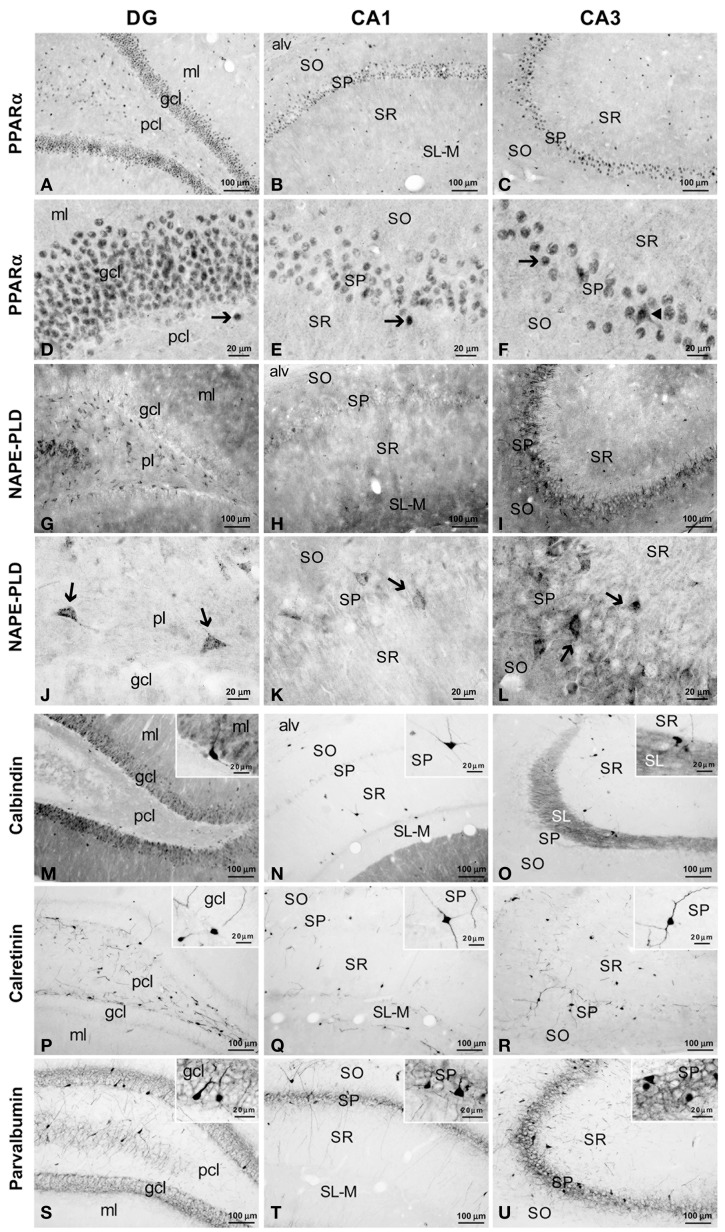
**Immunohistochemical expression of PPARα (A–F), NAPE-PLD (G–L) and the CaBPs calbindin (M–O), calretinin (P–R) and parvalbumin (S–U) in the medial rat hippocampus. (A–F)** Arrows indicate PPARα expression in the cell nuclei of the dentate granular, pyramidal, and non-pyramidal (interneurons) cells. The arrowhead in **(F)** indicates the cytoplasmic labeling of PPARα. **(G–L)** NAPE-PLD was associated with neuropil and puncta in the ml and gcl of the DG and the SO and SP of the CA1/3 fields. The arrows indicate the NAPE-PLD^+^ cells observed in the pcl of the DG and the SP of the CA1/3 fields. **(M–O)** Fibers in the ml of the DG and the SL of CA3 and cells in the gcl of the DG and the SO, SP, and SR of the CA1/3 fields were stained for calbindin. **(P–R)** A discrete number of cells in the pcl of the DG and all strata of the CA1/3 fields expressed calretinin. **(S–U)** Cells stained for parvalbumin were found in the gcl of the DG and the SP and SO of the CA1/3 fields, whereas fibers were observed in the gcl of the DG and the SP of the CA1/3 fields. For abbreviations, see Table 3 legend. Scale bars are indicated in each image.

To address the cell distribution of PPARα, NAPE-PLD and the CaBPs calbindin D28k, calretinin and parvalbumin in the rat hippocampus, coronal sections of the hippocampus were subjected to immunohistochemical analysis (Figure 2). Along the hippocampal complex, an intense and specific nuclear immunoreactivity for PPARα was detected in most cells localized in the granular cell layer (gcl) of the DG and stratum pyramidale (SP) of the CA1/3 fields (Figures 2A–F). Nuclear PPARα labeling was also observed in a number of non-principal cells widely distributed in the remaining layers and strata of the hippocampus, such as the molecular layer (ml) of the DG and the strata oriens (SO), radiatum (SR) and lacunosum-moleculare (SL-M) of the CA fields. Interestingly, it should be noted that some hippocampal cells in the SP showed additional staining for PPARα in their cytoplasm (Figure 2F).

NAPE-PLD immunoreactivity was specifically associated with neuropil and puncta in the ml and gcl of the DG, and the SO and SP of the CA1/3 fields (Figures 2G–I). In the SP of CA3, this neuropil surrounded the unstained profiles of the pyramidal cells. Weaker staining for NAPE-PLD was observed in puncta localized in the remaining hippocampal layers, and noteworthy staining was found in the basal part of the CA3 stratum radiatum, probably stratum lucidum (Figure 2I). Interestingly, a number of NAPE-PLD^+^ cells were observed in the dentate granular (gcl) and polymorphic (pcl or hilus) cell layers and the SP of the CA1/3 fields (Figures 2J–L). The remaining hippocampal layers showed a very small number of immunostained cells.

A large number of cells immunoreactive for calbindin D28k were detected in the gcl of the DG (Figure 2M). These calbindin^+^ cells showed moderate staining in comparison with the intense immunoreactivity observed in a few cells localized in the SO, SP, and SR of the CA1/3 fields (Figures 2M–O, insets). There appeared to be a slightly higher number of calbindin stained cells in the more temporal aspect of CA1. A high density of fibers was intensely stained for calbindin in the ml of the DG and the SL of CA3 (Figures 2M,O). Weaker calbindin staining was observed in the fibers of the pcl of the DG and in a band situated in the limit between the SR and SL-M of CA1 (Figures 2M,N).

Intense immunoreactivity for calretinin was associated with the somata and the proximal projections in a discrete number of cells localized in the pcl of the DG and all strata of the CA1/3 fields (Figures 2P–R, insets). A weak network of calretinin^+^ fibers was detected in the border between the gcl and ml of the DG (Figure 2P).

A discrete number of intensely stained cells positive for parvalbumin, which comprised the somata and proximal projections, was observed in the gcl of DG and the SP and SO of the CA1/3 fields (Figures 2S–U). Parvalbumin immunoreactivity was also associated with a dense meshwork of fibers localized in the gcl of the DG and the SP of the CA1/3 fields. These fibers surrounded the unstained profiles of the principal cells (Figures 2S–U, insets).

### Co-localization of PPARα and CaBPs in the adult rat hippocampus

**Figure 3:**
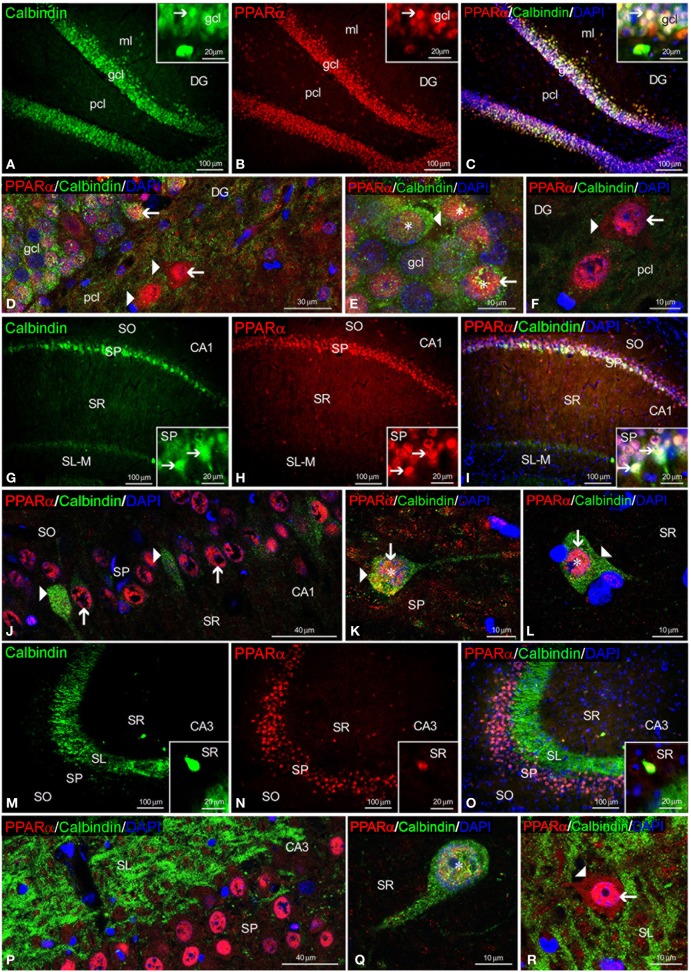
**PPARα and calbindin co-expression in the rat hippocampus.** Low-resolution epifluorescence photomicrographs **(A–C,G–I,M–O)** and high-resolution confocal laser scanning photomicrographs **(D–F,J–L,P–R)** showing the immunoreactivity of calbindin (green) and PPARα (red) in the dentate gyrus (DG), CA1, and CA3 areas. DAPI fluorescence (blue) was used to identify the cell nuclei of specific hippocampal cell populations. The arrows indicate PPARα expression in the cell nucleus. The arrowheads indicate the cytoplasmic labeling of PPARα or calbindin. The asterisks indicate examples of cells that co-express PPARα and calbindin. For abbreviations, see Table 3 legend. Scale bars are indicated in each image.

To study the co-expression of PPARα and the CaBPs calbindin D28k, calretinin and parvalbumin in the hippocampus, coronal sections were subjected to double-immunolabeling and confocal microscope analysis. DAPI immunofluorescence was used to determine the subcellular localization of PPARα and the CaBPs. PPARα and calbindin were highly co-expressed in most cell bodies of the gcl and ml of the DG (Figures 3A–E). Thus, the immunofluorescent signal of most PPARα^+^ cells (red) was localized in their cell nuclei, whereas the immunofluorescent signal of the calbindin^+^ cells (green) was mainly found in their cytoplasm (Figures 3D,E). In contrast, the PPARα^+^ cells in the pcl of the DG did not co-express calbindin, but they were surrounded by calbindin^+^ fibers (Figure 3F). Co-existence of PPARα and calbindin was observed in a small number of cells of the SP of CA1 but was not observed in the SP of CA3 (Figures 3G–L). Co-expression of PPARα and calbindin was scarce in the cells of the remaining strata in the CA1/3 fields (Figures 3M–Q). Interestingly, the intense network of calbindin^+^ fibers observed in the SL of CA3 was closely arranged around cells exhibiting PPARα immunofluorescence in the cell nucleus and cytoplasm (Figure 3R).

**Figure 4:**
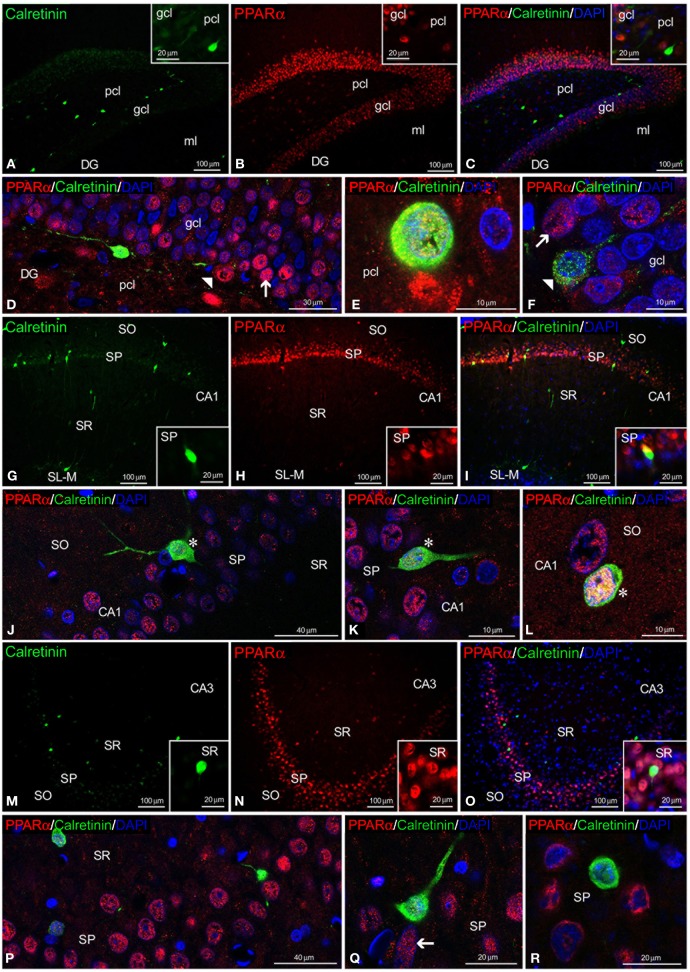
**PPARα and calretinin co-expression in the rat hippocampus.** Low-resolution epifluorescence photomicrographs **(A–C,G–I,M–O)** and high-resolution confocal laser scanning photomicrographs **(D–F,J–L,P–R)** showing the labeling of calretinin (green) and PPARα (red) in the dentate gyrus (DG), CA1, and CA3 areas. DAPI fluorescence (blue) is also shown. The arrows indicate PPARα expression in the cell nucleus. The arrowheads indicate the cytoplasmic labeling of calretinin. The asterisks indicate examples of cells that co-express PPARα and calretinin. For abbreviations, see Table 3 legend. Scale bars are indicated in each image.

**Figure 5:**
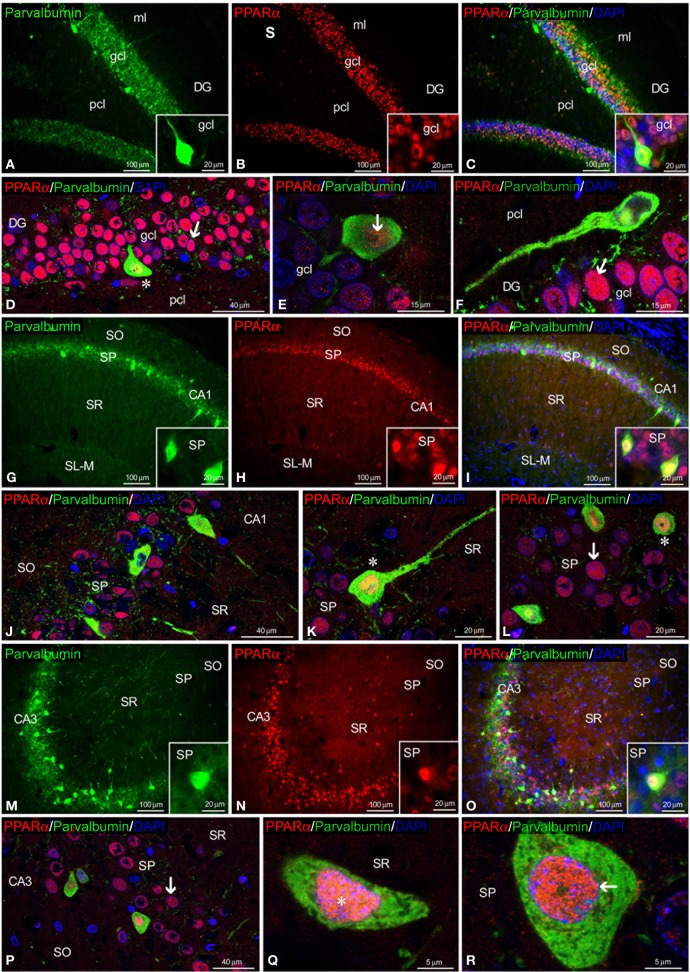
**PPARα and parvalbumin co-expression in the rat hippocampus.** Low-resolution epifluorescence photomicrographs **(A–C,G–I,M–O)** and high-resolution confocal laser scanning photomicrographs **(D–F,J–L,P–R)** showing the labeling of parvalbumin (green) and PPARα (red) in the dentate gyrus (DG), CA1, and CA3 areas. DAPI fluorescence (blue) is also shown. The arrows indicate PPARα expression in the cell nucleus. The asterisks indicate examples of cells that co-express PPARα and parvalbumin. For abbreviations, see Table 3 legend. Scale bars are indicated in each image.

Regarding calretinin and parvalbumin expression, co-expression in PPARα^+^ cells was scarce in the rat hippocampus (Figures 4, 5). PPARα and calretinin were co-expressed in some cells of the pcl of the DG (Figures 4A–F), the SO, SP, and SR of CA1 (Figures 4G–L) and the SO and SP of CA3 (Figures 4M–R). PPARα^+^/parvalbumin^+^ cells were mainly localized in the inner border of the gcl of the DG (Figures 5A–F, insets), the SP of CA1 (Figures 5G–L, insets) and the SP and SR of CA3 (Figures 5M–R, insets). In all cases, similar to calbindin, both calretinin and parvalbumin were localized in the cytoplasm (Figures 5D,K,Q,R). The meshwork of parvalbumin^+^ fibers localized in the gcl of the DG and the SP of the CA1/3 fields surrounded the PPARα^+^ cells (Figures 5D,J,P).

### Co-localization of NAPE-PLD and CaBPs in the adult rat hippocampus

**Figure 6:**
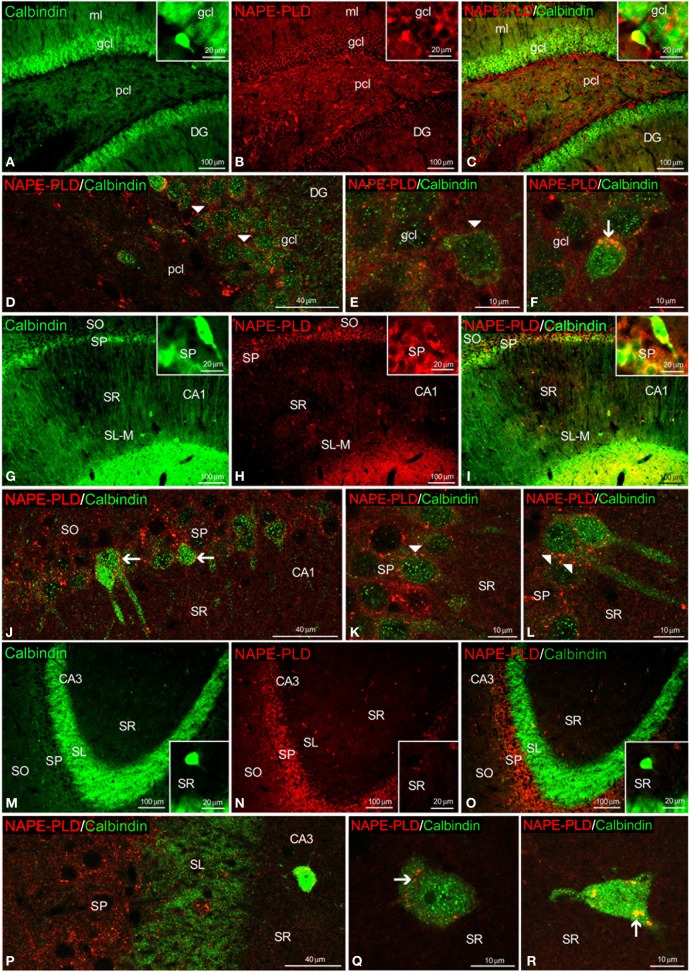
**NAPE-PLD and calbindin co-expression in the rat hippocampus.** Low-resolution epifluorescence photomicrographs **(A–C,G–I,M–O)** and high-resolution confocal laser scanning photomicrographs **(D–F,J–L,P–R)** showing the labeling of calbindin (green) and NAPE-PLD (red) in the dentate gyrus (DG), CA1, and CA3 areas. The arrows indicate NAPE-PLD expression in the calbindin^+^ cells. The arrowheads indicate the NAPE-PLD^+^ fiber varicosities on the surface of the calbindin^+^ cells. For abbreviations, see Table 3 legend. Scale bars are indicated in each image.

To study the co-existence of NAPE-PLD and the CaBPs calbindin D28k, calretinin and parvalbumin in the hippocampus, coronal sections were also subjected to double-immunolabeling and confocal microscope analysis. The high number of calbindin^+^ cells localized in the gcl of the DG was surrounded by a meshwork of NAPE-PLD^+^ neuropil and puncta (Figures 6A–E). We could also find co-expression of NAPE-PLD and calbindin in some cells of the gcl of the DG (Figure 6F). In the SP of CA1, NAPE-PLD^+^ neuropil and puncta also surrounded some calbindin^+^ cells and a number of unstained profiles of pyramidal cells (Figures 6G–L). In contrast, we could not find NAPE-PLD^+^ puncta surrounding calbindin^+^ cells in the SP of CA3 (Figures 6M–P), but we observed NAPE-PLD labeling in the somata of the calbindin^+^ cells in the SR of CA3 (Figures 6Q,R).

**Figure 7:**
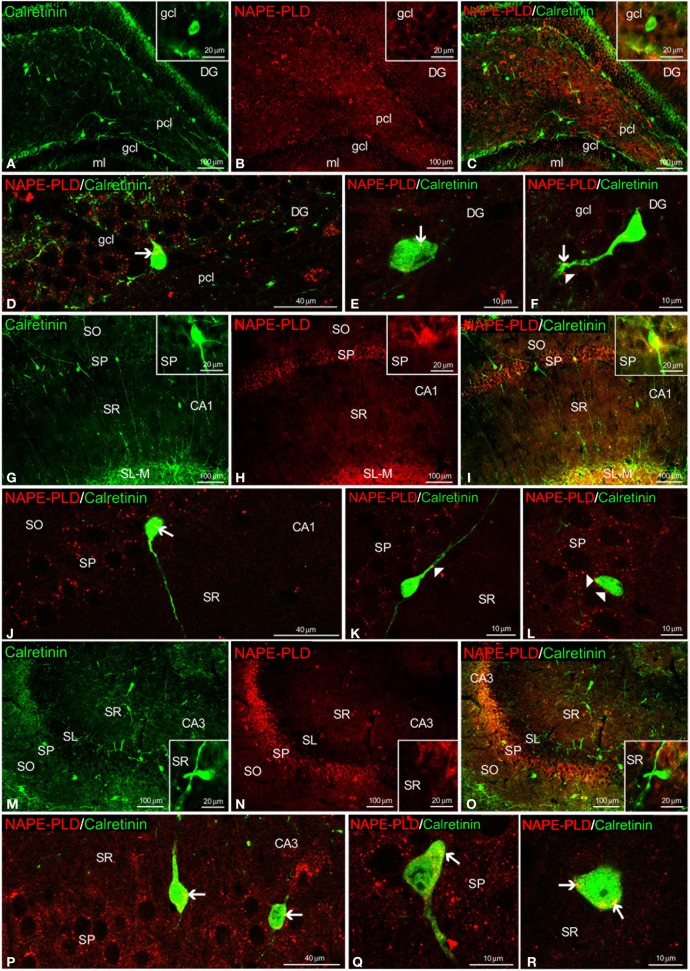
**NAPE-PLD and calretinin co-expression in the rat hippocampus.** Low-resolution epifluorescence photomicrographs **(A–C,G–I,M–O)** and high-resolution confocal laser scanning photomicrographs **(D–F,J–L,P–R)** showing the labeling of calretinin (green) and NAPE-PLD (red) in the dentate gyrus (DG), CA1, and CA3 areas. The arrows indicate NAPE-PLD expression in the calretinin^+^ cells. The arrowheads indicate the NAPE-PLD^+^ fiber varicosities on the surface of the calretinin^+^ cells. For abbreviations, see Table 3 legend. Scale bars are indicated in each image.

We also observed NAPE-PLD labeling in the somata and proximal axons of the calretinin^+^ cells localized in the gcl of the DG (Figures 7A–E) and the SP and SR of the CA1/3 fields (Figures 7G–J,M–R). NAPE-PLD^+^ puncta were detected on the surface of some somata, as well as in the proximal axons, of cells in the gcl of the DG (Figure 7F) and the SP of CA1 (Figures 7K,L).

**Figure 8:**
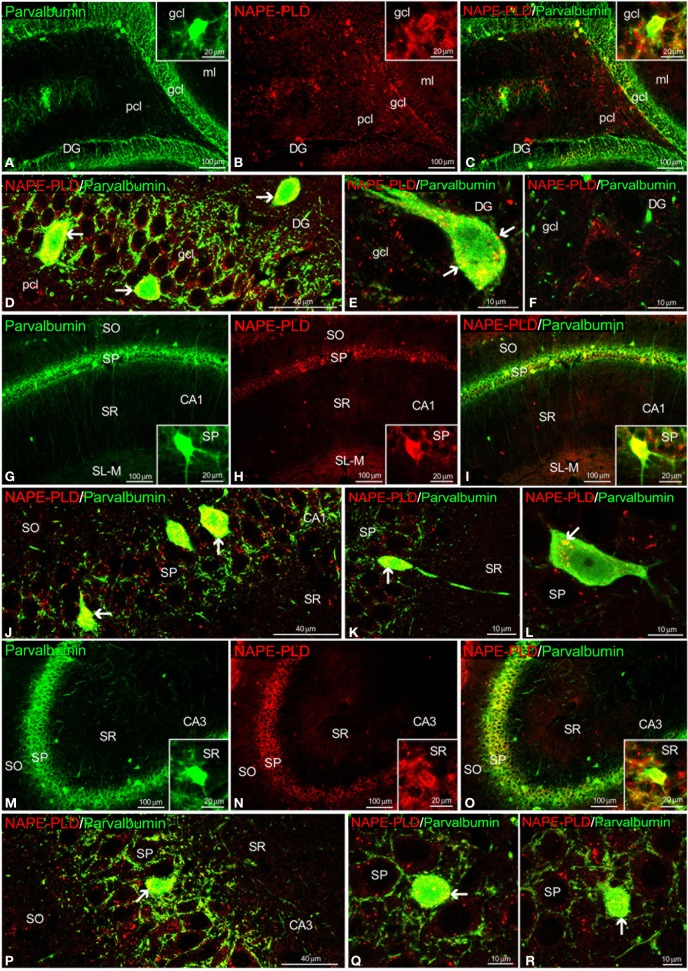
**NAPE-PLD and parvalbumin co-expression in the rat hippocampus.** Low-resolution epifluorescence photomicrographs **(A–C,G–I,M–O)** and high-resolution confocal laser scanning photomicrographs **(D–F,J–L,P–R)** showing the labeling of parvalbumin (green) and NAPE-PLD (red) in the dentate gyrus (DG), CA1, and CA3 areas. The arrows indicate NAPE-PLD expression in the parvalbumin^+^ cells. For abbreviations, see Table 3 legend. Scale bars are indicated in each image.

Most NAPE-PLD^+^ cells localized in the gcl and pcl of the DG and the SP of the CA1/3 fields also exhibited parvalbumin expression (Figures 8A–E,G–R). Some NAPE-PLD^+^/parvalbumin^−^ cells in the gcl of the DG were surrounded by parvalbumin^+^ puncta (Figure 8F). Moreover, the unstained profiles of granular cells in the DG and the pyramidal cells in the CA fields were surrounded by parvalbumin^+^ fibers and puncta that were closely intercalated with NAPE-PLD^+^ puncta (Figures 8D,J,P).

### Quantification of co-localization in the adult rat hippocampus

**Figure 9:**
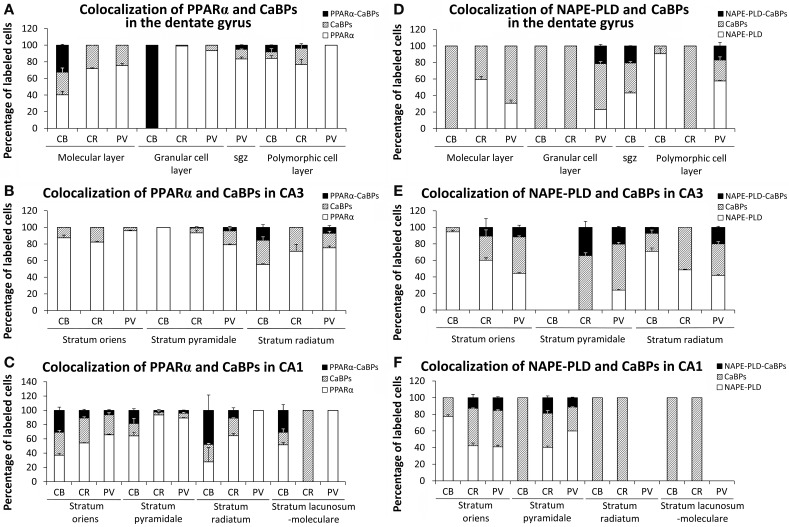
**Quantification of the proportion of cells labeled with PPARα (A–C) or NAPE-PLD (D–F) that express calbindin, calretinin, or parvalbumin in each layer of the dentate gyrus, CA3, and CA1.** Bars represent the percentage of labeled cells in each layer.

We quantified the proportion of PPARα or NAPE-PLD-labeled cells that express calbindin, calretinin or parvalbumin in each layer of the DG, CA3, and CA1 (Figure 9). All PPARα-labeled cells express calbindin in the granular cell layer of the DG. A lower proportion of PPARα-calbindin-labeled cells was also found in the molecular (32.2%) and polymorphic (7.9%) cell layers of the DG (Figure 9A), SR of CA3 (15.4%) (Figure 9B), and SO (30.5%), SP (18.4%), SR (39.6%), and SL-M (29.65) of CA1 (Figure 9C). We observed a low percentage of cells that express PPARα and calretinin in the polymorphic cell layer of the DG (3.7%) (Figure 9A), and SO (10.5%), SP (2.3%), and SR (11.2%) of CA1 (Figure 9C). We also observed a very low percentage of cells that express PPARα and parvalbumin in the subgranular zone (sgz) of the DG (5.1%) (Figure 9A), SP (4.3%) and SR (7.1%) of CA3 (Figure 9B), and SO (6.3%) and SP (3.9%) of CA1 (Figure 9C). A proportion of NAPE-PLD-calbindin-labeled cells was obtained in the sgz of the DG (20.5%) (Figure 9D), and SR of CA3 (7.2%) (Figure 9E). We also observed a representative percentage of NAPE-PLD-labeled cells that express parvalbumin in the granular (21.1%) and polymorphic (16.9%) cell layers of the DG (Figure 9D). Finally, a percentage of NAPE-PLD-labeled cells expressing parvalbumin or calretinin was observed in SO (11.6 and 10.7%, respectively), SP (20.3 and 32.9%, respectively) and SR (20.1%) of CA3 (Figure 9E), and SO (15.4 and 12.8%, respectively) and (11.3 and 18.8%, respectively) SP of CA1 (Figure 9F).

## Discussion

**Figure 10:**
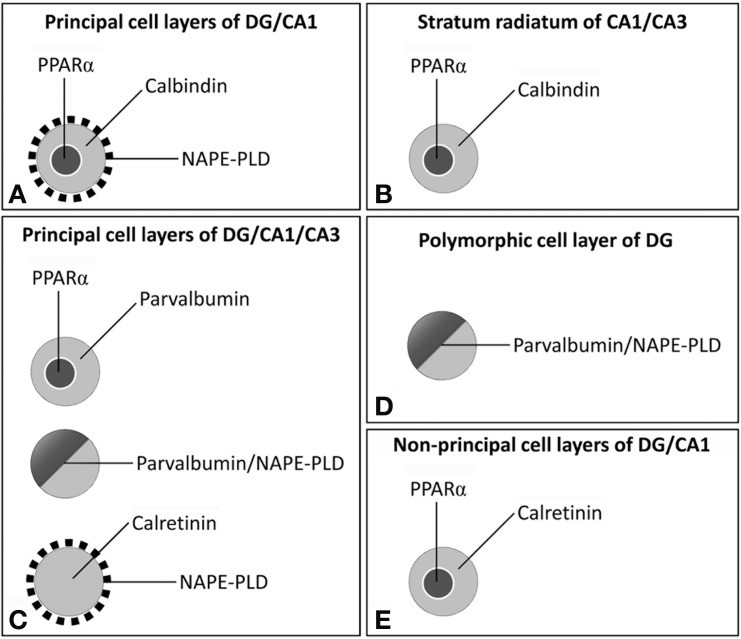
**Schematic representation of the main cell populations containing PPARα /NAPE-PLD that expressed the CaBPs calbindin, calretinin, and parvalbumin in the different hippocampal regions. (A)** Most dentate granular cells and some CA1 pyramidal cells co-expressed calbindin and nuclear PPARα and were closely surrounded by NAPE-PLD^+^ fiber varicosities. **(B)** Cells expressing both calbindin and PPARα were also found in the SR of CA1/3. **(C)** In the principal cell layers of the DG and the CA1/3 fields, most cells containing parvalbumin expressed both NAPE-PLD and PPARα, and the cell bodies that expressed calretinin, but most likely not PPARα, were surrounded by NAPE-PLD^+^ puncta. **(D)** Cells expressing both parvalbumin and NAPE-PLD were also found in the pcl of the DG. **(E)** A small number of PPARα^+^ cell bodies expressed calretinin, mainly in the non-principal cell layers of the hippocampus.

The biological activities of monounsaturated NAEs require both release from membrane precursors by the Ca^2+^-dependent NAPE-PLD and the activation of PPARα receptors that modulate Ca^2+^-dependent mechanisms. Thus, the identification of specific populations of NAPE-PLD/PPARα-containing neurons that express selective Ca^2+^-binding proteins (CaBPs) may provide a neuroanatomical basis to better understand the NAEs/PPARα signaling system in the brain. Our study provides evidence for an anatomical segregation of the PPARα signaling system in hippocampal cells by identifying the localization and co-existence of PPARα, NAPE-PLD, and the CaBPs calbindin, calretinin and parvalbumin, which have not been previously described. Due to the specific localization of PPARα in the cell nuclei, NAPE-PLD in particular puncta and cells, and CaBPs in certain cell bodies and fibers, we were able to highlight at least 4 different cell populations in the rat hippocampus (Figure 10). First, we observed that most dentate granular cells and some CA1 pyramidal cells co-expressed both calbindin and nuclear PPARα. These calbindin^+^ cells containing PPARα were closely surrounded by NAPE-PLD^+^ fiber varicosities (Figure 10A). Cells expressing both calbindin and PPARα were also found in the SR of CA1/3 (Figure 10B). Second, we observed that most cells containing parvalbumin expressed both NAPE-PLD and PPARα in the principal cell layers of the DG and CA1/3 fields (Figure 10C). Cells expressing both parvalbumin and NAPE-PLD were also found in the pcl of the DG (Figure 10D). Third, cell bodies that expressed calretinin, but most likely not PPARα, were also surrounded by NAPE-PLD^+^ puncta in the principal cell layers of the DG and CA1/3 fields (Figure 10C). Fourth, a small number of PPARα^+^ cell bodies expressed calretinin, mainly in the non-principal cell layers of the hippocampus (Figure 9E).

We observed that NAPE-PLD immunostaining was completely absent in the NAPE-PLD knock-out mouse hippocampus (Leung et al., 2006; Nyilas et al., 2008), whereas the wild type mouse showed similar labeling to that described by Egertová et al. (2008). Thus, in the mouse hippocampus, very intense NAPE-PLD immunoreactivity was localized in the mossy fibers of the polymorphic cell layer of the DG and the stratum lucidum of CA3. Weaker staining was also observed in the molecular layer of the DG and the strata oriens and radiatum of the CA fields. In contrast to Egertová et al. (2008), we observed a number of NAPE-PLD^+^ cells in the granular and polymorphic cell layers of the DG and the stratum pyramidale of the CA fields in the mouse hippocampus, as well as in the rat hippocampus. No stained cells were detected in the hippocampus of the NAPE-PLD knock-out mouse. Additionally, some differences in NAPE-PLD immunostaining were found when the rat and mouse hippocampi were compared. Thus, it should be noted a weaker NAPE-PLD immunoreactivity in the basal part of the CA3 stratum radiatum in the rat hippocampus. On the contrary, a high density of NAPE-PLD^+^ neuropil and puncta were observed in the stratum pyramidale of the CA fields, but not in the dentate polymorphic cell layer. These fibers surrounded the unstained cells, similar to those described in the polymorphic cell layer of the mouse hippocampus (Egertová et al., 2008). These results suggest specie-specific differences, at least at protein level of NAPE-PLD, between the rat and mouse hippocampi.

Our results confirm data from previous studies on the presence and localization of PPARα in the rat hippocampus (Cullingford et al., 1998; Moreno et al., 2004). However, Moreno and collaborators showed PPARα immunoreactivity in both nuclear and cytoplasmic localizations, whereas we observed PPARα expression specifically in the cell nucleus and, occasionally, in the cytoplasm of scattered cells. Calbindin immunoreactivity was described in the granular cells of the DG, some pyramidal cells of CA1 and scattered interneurons of the SR of hippocampus (Baimbridge and Miller, 1982). In the present study, we observed that calbindin^+^ cells expressed nuclear PPARα in the principal cell layers of the DG and CA1 and in the stratum radiatum of CA1/3. The presence of PPARα^+^/calbindin^+^ cells in specific localizations of the hippocampus provides neuroanatomical evidence of the relationship between the neuroprotective role of PPARα in neurodegenerative disorders (Aleshin et al., 2013) and the dysregulation of neuronal calcium homeostasis, such as in calbindin-deficient mice, which have impaired long-term hippocampal potentiation and thus impaired spatial learning and memory functioning (Molinari et al., 1996; Hajieva et al., 2009; Soontornniyomkij et al., 2012). For instance, the over-expression of calbindin selectively in the dentate granule cells disrupted the presynaptic function of the mossy fibers, reduced long-term potentiation and impaired spatial memory (Dumas et al., 2004). It is interestingly to note that no PPARα^+^/calbindin^+^ cells were detected in the stratum pyramidale of CA3, and the pyramidal cells of CA3, which did not contain PPARαand calbindin, are one of the cell types that are most susceptible to burst discharge and epileptiform activity (Kandel and Spencer, 1961).

Parvalbumin^+^ cells were specifically localized in the granular and polymorphic cell layers of the DG and the strata oriens and pyramidale in the CA1/3 fields of the rat hippocampus (Kosaka et al., 1987). These cells have been considered a subpopulation of GABAergic interneurons, including the basket and axo-axonic cell types. In the present study, we described PPARα^+^/parvalbumin^+^ cells and NAPE-PLD^+^/parvalbumin^+^ cells that were specifically concentrated in the granular cell layer of the DG and the stratum pyramidale of the CA1/3 fields. Based on their morphology and localization in the hippocampal layers, it is reasonable to suggest that PPARα and NAPE-PLD are expressed in the same parvalbumin cell population (i.e., basket cells). Moreover, based on the physiological characteristics of the neurons with parvalbumin content (Heizmann, 1984) and their important role in the synchronization of action potential discharges (Freund, 2003), we hypothesize that the PPARα/NAPE-PLD/parvalbumin-containing GABAergic neurons may be characterized by their high spontaneous discharge rate and high firing rate in response to depolarizing currents, at least in the hippocampus (Schwartzkroin and Kunkel, 1985; Celio, 1986; Kosaka et al., 1987). Thus, it is conceivable to think that impairment of endocannabinoid tone in the hippocampal basket cells is likely to result in alteration in cognitive processes and mood disorders such as anxiety (Busquets-Garcia et al., 2011). The presence of PPARα^+^/parvalbumin^+^ cells in specific localizations of the hippocampus can provide other neuroanatomical evidence regarding the relationship between the neuroprotective role of PPARα in the prevention of calcium influx induced by H_2_O_2_ and the enhancement of calcium resequestration by parvalbumin and its implications in energy metabolism (Santos et al., 2005; Seebacher and Walter, 2012).

Previous observations in the rat hippocampus indicated that calretinin-containing neurons were heterogeneous both morphologically and neurochemically (Gulyás et al., 1992; Miettinen et al., 1992). Calretinin immunoreactivity was almost exclusively described in GABAergic non-pyramidal cells in the layers of the DG and the CA1/3 fields, being most abundant in the polymorphic cell layer of the DG and the stratum lucidum of CA3. However, GABA-negative calretinin-containing neurons that are specifically localized in the polymorphic cell layer of the DG and the stratum lucidum of CA3 have been described (Miettinen et al., 1992). In the present study, we observed that very few calretinin^+^ cells expressed PPARα in the hippocampus; most of the calretinin^+^ cells were in the polymorphic cell layer of the DG and the stratum oriens of CA3. Thus, further analysis is necessary to determine whether PPARα^+^/calretinin^+^ cells are GABAergic neurons, at least in the polymorphic cell layer of the DG.

In conclusion, our data indicated the presence of hippocampal cell subpopulations with specific co-expression patterns of the NAPE-PLD/PPARα system and selective CaBPs. As the CaBPs calbindin, calretinin and parvalbumin are found in a limited number of hippocampal cell types that express PPARα and NAPE-PLD, we suggest that these CaBPs-expressing cells, especially those expressing calbindin and parvalbumin, may be involved in more specialized Ca^2+^-regulated functions related to the biological roles of the PPARα signaling system.

## Author contributions

All authors had full access to all the data in the study and take responsibility for the integrity of the data and the accuracy of the data analysis. Study concept and design: Fernando Rodríguez de Fonseca and Juan Suárez. Acquisition of the data: Patricia Rivera, Sergio Arrabal, Eduardo Blanco, Antonia Serrano, and Francisco J. Pavón. Analysis and interpretation of the data: Patricia Rivera and Juan Suárez. Drafting of the manuscript: Patricia Rivera and Juan Suárez. Critical revision of the manuscript for important intellectual content, obtained funding and study supervision: Fernando Rodríguez de Fonseca and Juan Suárez.

### Conflict of interest statement

The authors declare that the research was conducted in the absence of any commercial or financial relationships that could be construed as a potential conflict of interest.
